# Salt-inducible kinases (SIK) inhibition reduces RANKL-induced osteoclastogenesis

**DOI:** 10.1371/journal.pone.0185426

**Published:** 2017-10-03

**Authors:** Maria Stella Lombardi, Corine Gilliéron, Majoska Berkelaar, Cem Gabay

**Affiliations:** 1 Division of Rheumatology, Department of Internal Medicine Specialties, University Hospitals of Geneva, Geneva, Switzerland; 2 Department of Pathology and Immunology, University of Geneva School of Medicine, Geneva, Switzerland; Charles P. Darby Children's Research Institute, 173 Ashley Avenue, Charleston, SC 29425, USA, UNITED STATES

## Abstract

Osteoclasts are large multinucleated cells responsible for bone resorption. Excessive inflammatory activation of osteoclasts leads to bony erosions, which are the hallmark of several diseases such as rheumatoid arthritis (RA). Salt-inducible kinases (SIK) constitute a subfamily of kinases comprising three members (SIK1, -2, and -3). Inhibition of SIK kinase activity induces an anti-inflammatory phenotype in macrophages. Since osteoclasts originate from precursors of macrophage origin, we hypothesized a role of SIK in osteoclastogenesis. We analyzed SIK1, -2 and -3 expression and function in osteoclast differentiation using the mouse macrophage cell line RAW264.7 and bone marrow-derived macrophages (BMM). We show that all three SIK are expressed in fully differentiated osteoclasts and that in BMM-derived osteoclasts there is an increased expression of SIK1 and SIK3 proteins. Interestingly, the pan-SIK inhibitor HG-9-91-01 significantly inhibited osteoclastogenesis by dose dependently reducing osteoclast differentiation markers (i.e. CathepsinK, MMP-9 and TRAP) and bone resorbing activity. Analysis of the signaling pathways activated by RANKL in RAW cells showed that SIK inhibitors did not affect RANKL-induced ERK1/2, JNK, p38 or NF-κB activation, but induced a significant downregulation in c-Fos and NFATc1 protein levels, the two main transcription factors involved in the regulation of osteoclast-specific genes. Moreover, SIK inhibition partially increased the proteasome-mediated degradation of c-Fos. SIK2 and SIK3 knockout RAW cells were generated by the CRISPR/Cas9 approach. SIK2 KO and, to a lesser extent, SIK3 KO recapitulated the effect of SIK small molecule inhibitor, thus confirming the specificity of the effect of SIK inhibition on the reduction of osteoclastogenesis.

Overall, our results support the notion that the SIK signaling pathway plays a significant role among the check-points controlling osteoclastogenesis. SIK kinase inhibitors could thus represent a potential novel therapy to prevent bone erosions.

## Introduction

Salt-inducible kinases (SIK) constitute a serine/threonine kinase subfamily that belongs to the AMP-activated protein kinase (AMPK) family. Three members: SIK1, 2 and 3 have been identified so far [1]. SIK are involved in the modulation of toll-like receptor (TLR)-induced pro-inflammatory signals. Indeed, the function of SIKs in macrophages is to restrict the formation of regulatory phenotypes by limiting the production of anti-inflammatory cytokines (e.g. IL-10) [2]. Consequently, upon TLR stimulation the inhibition of SIKs by a small molecule kinase inhibitor induces a skewing to an anti-inflammatory phenotype characterized by very low IL-12/TNFα, significantly reduced IL-6 and IL-1β and increased IL-10 production [3]. Mechanistic studies using the selective pan-SIK inhibitor HG-9-91-01 in mouse bone-marrow derived macrophages (BMDM) revealed that the downstream effects observed with SIK inhibitors on cytokine modulation correlated with dephosphorylation and consequent nuclear translocation in these cells of two direct SIK targets: CREB-regulated transcription coactivator-3 (CRTC3) and histone deacetylase 4 (HDAC4) [2, 4]. We recently confirmed these observations in TLR-stimulated human myeloid cells (macrophages and dendritic cells) and in addition demonstrated for the first time that SIK inhibition by HG-9-91-01 decreases pro-inflammatory cytokines in human myeloid cells also upon IL-1R stimulation [5]. Our data expanded the potential therapeutic use of SIK inhibitors in immune-mediated inflammatory diseases.

However, it is not known whether SIK could play additional roles in other cell types such as osteoclasts that are relevant for inflammatory diseases such as rheumatoid arthritis (RA). Osteoclasts are giant multinucleated bone-resorbing cells derived from the fusion of precursor macrophages under the effect of M-CSF and RANKL stimulation [6, 7]. The binding of RANKL to its receptor RANK leads to recruitment of TNF-receptor associated factor 6 (TRAF6), which in turns triggers various signalling pathways such as NF-κB [8] as well as the three mitogen-activated protein kinases p38 MAPK, JNK and ERK [9, 10]. Given that RANK and TLR share some common signalling molecules [3] we hypothesized that SIK could possibly play a role in RANKL-mediated signalling in osteoclastogenesis. By using RANKL-stimulated RAW264.7 mouse cells or bone marrow-derived mouse macrophages (BMM), we examined the expression and function of SIK proteins in osteoclastogenesis.

## Materials and methods

### Drugs and reagents

HG-9-91-01 was synthesized as described elsewhere [2] and purified to > 96% purity by Syngene International (Bangalore, India). Powder was dissolved in DMSO (Hybri-MAX, Sigma-Aldrich, St. Louis, MO, USA) as 10 mM stock solutions and stored at -20°C until use. Recombinant mouse RANKL was from R&D Systems (Minneapolis, MN, USA). Recombinant mouse M-CSF (rmM-CSF) was from ImmunoTools (Friesoythe, Germany). BSmBI restriction enzyme was from Thermo Fisher Scientific, (Waltham, MA, USA) pLentiCRISPR-V2 (GeCKO), pVSVg (AddGene#8454) and psPAX2 (AddGene #12260) plasmids were a kind gift of Dr. Fabio Martinon (Unil, Lausanne, Switzerland).

### Cell culture

The murine monocyte/macrophage cell line RAW 264.7 was purchased from American Type Culture Collection (Manassas, VA, USA) and grown in DMEM supplemented with 2mM L-glutamine,100U/ml penicillin/streptomycin (all from GIBCO, New York, NY, USA) and 10% heat-inactivated FBS (GIBCO, New York, NY, USA). Cells were grown in a humidified atmosphere containing 5% CO_2_ at 37°C.

For osteoclast differentiation, RAW 264.7 cells were suspended in α-MEM (GIBCO, New York, NY, USA) containing 5% FBS, 2mM L-glutamine, 100U/ml penicillin/streptomycin and then seeded at 25 x 10^3^ cells/well in 24-well culture plates in presence of 50 ng/ml rmRANKL for 4 days. The medium was replaced every 2 days.

Isolation of bone marrow cells from C57BL/6 mice was performed in mice sacrificed by CO_2_ asphyxiation without any further experimental manipulation under the authorization of the Geneva cantonal authority for animal experimentation (license No GE5-15). These experiments were carried out as previously described [5]. Briefly, bone marrow cells were cultured in α-MEM containing 10% FBS and 30 ng/ml rmM-CSF for 24h in bacterial culture grade 10 mm Petri dishes. Non-adherent cells were harvested and cultured for 3-days in tissue culture-treated plates in presence rmM-CSF (30ng/ml). Floating cells were then removed and adherent cells (bone marrow derived macrophages, BMM) were used as osteoclast precursors. To generate osteoclasts, BMM were seeded at 160 x 10^3^ cells/well in a 24 well culture plate and further cultured with rmM-CSF (30ng/ml) and rmRANKL (50ng/ml) for 3 days.

### TRAP staining

For the TRAP staining, RAW 264.7 cells were seeded at 25 x 10^3^ cells/well in a 24 well plate for 4 days and BMM were seeded at 160 x10^3^ cells/well in a 24 well plate for 3 days. Briefly, the cells were washed with PBS and fixed with 3.7% formaldehyde for 30 sec. After washing with PBS, cells were stained for 30 min at 37°C in the dark with a mixture of solutions of Fast Garnet GBC, sodium nitrite, naphtol AS-BI acid phosphoric, acetate and tartrate of the Leukocyte Acid Phosphatase Assay Kit (Sigma-Aldrich, St. Louis, MO, USA) following manufacturer’s instructions. TRAP- positive multinucleated cells (MNCs) containing 3 or more nuclei were counted as osteoclasts.

### Resorption lacunae assay

BMM cells were plated at concentration of 160 x 10^3^ cells/well in 24 wells osteo assay plates coated with bone biomimetic synthetic surface (ref, #3987, Corning^®^, Kennebunk ME, USA) and cultured with rmM-CSF (30ng/ml) and rmRANKL (50ng/ml) for 3 days in absence or presence of different concentrations of HG-9-91-01. At the end of the culture, plates were washed twice with PBS 1X. Subsequently, 400μl/well of 5% sodium hypochlorite solution were added for 10 min at R.T. to remove the cells, the wells were washed 3 times with water and dried overnight at R.T. The resorbed areas on the plates were captured with a digital camera using a Zoe Cell Imager (Bio-Rad) and analyzed by Metamorph Green imaging analysis (Metamorph imaging system, Downingtown, PA, USA). The resorption area was expressed as percentage of the RANKL-treated alone group.

### RNA extraction, reverse transcription and real-time PCR analysis

Total RNAs were extracted from cultured cells using TRIZOL (Ambion, Austin, TE, USA) following manufacturer’s instructions. Complementary DNA was generated from 1μg of DNAse RQ1-treated total RNA in a 20μl reaction with SuperScript II reverse transcriptase (Invitrogen, Carlsbad, CA, USA) following the manufacturer’s instructions and using random hexamer. Real time qPCR (40 cycles, annealing temperature 60°C) was performed using a master mix (SYBR green Supermix, Bio-Rad, Hercules, CA, USA) on a CFX96 real-time system (Bio-Rad). The primers used for qPCR are listed in Table 1. Relative expression of each gene was calculated from *Ct* values using Pfaffl method [11] and normalized against the 18S rRNA.

**Table 1:** Oligonucleotides used for real-time PCR analysis.

Gene	Forward sequence	Reverse sequence
**Cathepsin K**	5’-CACCCAGTGGGAGCTATGGAA-3’	5’-GCCTCCAGGTTATGGGCAGA-3’
**MMP9**	5’-GCCCTGGAACTCACACGACA-3’	5’-TTGGAAACTCACACGCCAGAAG
**TRAP**	5’-TCCTGGCTCAAAAAGCAGT-3’	5’-ACATAGCCCACACCGTTCTC-3’
**c-Fos**	5’-ACCGGTCCACAGAGGTTCAT-3’	5’-GCCTCTCGGAGTCTGGTCTT-3’
**NFATc1**	5’-GGTGGCCTCGAACCCTATC-3’	5’-TCAGTCTTTGCTTCCATCTCCC-3’
**SIK1**	5’-TCCAGACCATCTTGGGGCAG-3’	5’-AAGGGGAAGGGGTTTTGTGTTG-3’
**SIK2**	5’-GGGTGGGGTTCTACGACATC-3’	5’-TATTGCCACCTCCGTCTTGG-3’
**SIK3**	5’-CTCAGCCATCTCCACCTCTTCA-3’	5’-GGCTGCCTGAAGAGATGGTTGT-3’
**18S rRNA**	5′-GTAACCCGTTGAACCCCATT-3’	5′-CCATCCAATCGGTAGTAGCG-3’

### Immunoblotting

Cells were rinsed once in ice-cold 1X PBS and extracted in Cell Lysis Buffer (20 mM Tris-HCl, pH 7.5, 150 mM NaCl, 1mM EDTA, 1mM EGTA, 1% (v/v) Triton X-100) supplemented with 1X Complete EDTA-free protease inhibitor mixture and 1X PhosSTOP phosphatase inhibitor (Roche, Basel, Switzerland). Cell extracts were clarified by centrifugation at 14,000 x*g* for 15 min at 4°C. For nuclear and cytosolic proteins separation the cell pellet (obtained from ~ 7.5x10^6^ cells) was suspended in 400 μl of Hypotonic Lysis Buffer (10 mM Hepes pH 7.9, 10 mM KCl, 0.1 mM EGTA, 0.1 mM EDTA, 1 mM DTT, supplemented with protease and phosphatase inhibitors as above) and incubated on ice for 15min. After addition of 0.125% of NP-40 and vortexing for 10 sec, nuclei were sedimented by centrifugation at 15.000x *g* for 5 min at 4°C. The supernatant (cytosolic fraction) was transferred in new tubes. The pellet was resuspended in 50–100 μl of Nuclear Extraction Buffer (20 mM Hepes pH 7.9, 0.4 mM NaCl, 1 mM EGTA, 1 mM EDTA, 1 mM DTT, supplemented with protease and phosphatase inhibitors as above), rocked for 15 min at 4°C and clarified by centrifugation as above. The supernatant represented the nuclear protein fraction. All extracts were snap-frozen in liquid nitrogen and kept at -80°C until use. Protein concentration was determined using the Bradford assay (Bio-Rad, Hercules, California USA) and 10–25 μg of total cell extracts (or 30–50 μg for nuclear cell extracts) were separated by SDS/PAGE using 7.5% or 10% Acrylamide gel and transferred to nitrocellulose membranes. Immunoreactive bands were visualized by ECL reagents (Amersham biosciences, Buckinghamshire, UK) or Radiance Plus (Axonlab, Baden, CH) and signals acquired using a LAS4000 mini imager (Fujifilm Life science, Stamford, CT, USA) and quantified using ImageJ software 1.47v (NIH). Membranes were stripped in 1X Re-blot Plus Strong (Millipore, Billerica, MA, USA) following manufacturer’s instructions.

### CRISPR/Cas9 mediated knock out of SIK in RAW264.7 cells

CRISPR sequences targeting exon 2 of mouse *SIK1*, exon 1 of mouse *SIK2* and exon 4 of mouse *SIK3* were designed using the online available CRISPR design tool developed by the F. Zhang laboratory (http://crispr.mit.edu/) and the corresponding oligonucleotides were synthesized (Eurofins Genomics, Ebersberg, Germany). The seed sequences preceding the protospacer adjacent motif (PAM) are listed in Table 2. Nucleotides in italics show the overhangs necessary for incorporation into the restriction enzymatic site BsmBI of pLentiCRISPR-V2 vector expressing Cas9 (Addgene #52961). The vector was digested with BsmBI, and the annealed oligos were cloned according to the protocol from Zhang laboratory [12]. Correctness of the insertion was verified by Sanger sequencing (Fasteris, Geneva, Switzerland). To produce the lentivirus, pLentiCRISPR-V2 (with cloned sgRNA) were co-transfected into HEK293T cells with the packaging plasmids pVSVg (AddGene#8454) and psPAX2 (AddGene #12260) as previously described [13]. RAW264.7 cells were then infected with pLentiCRISPR-V2 viruses targeting SIK1, SIK2, and SIK3, respectively in presence of 8 μg/ml polybrene. Two days after infection, positive cells were selected for two weeks with 3 μg/mL puromycin (Santa Cruz Biotechnology, Dallas, TX, USA) as determined previously by a puromycin titration experiment in RAW264.7 cells. SIK expression was assessed by immunoblotting. A detailed description can be found in Supplementary Methods.

**Table 2:** Oligonucleotides used for CISPR/Cas9 gene editing in murine RAW 264.7cells, in italics recognition sequence of BsmBI.

Oligo name	Oligo sequence 5’ -> 3’
gRNA SIK1_1	5’-*CACCG***ACGGGCACTTGAGTGAAAAC**-3’
gRNA SIK1_2	5’-*AAAC***GTTTTCACTCAAGTGCCCGT***C*-3’
gRNA SIK2_1	5’-*CACCG***CTACGACATCGAGGGCACGC**-3’
gRNA SIK2_2	5’-*AAAC***GCGTGCCCTCGATGTCGTAG***C*-3’
gRNA SIK3_1	5’-*CACCG***GTTCAAACAGATCGTCACAG**-3’
gRNA SIK3_2	5’-*AAAC***CTGTGACGATCTGTTTGAAC***C*-3’

### Antibodies

The following antibodies were used for immunoblotting: anti-mouse, anti-rabbit HRP-conjugated secondary antibodies, anti NFATc1 (H110) and anti-α-Tubulin (Sc-23948) all from (Santa Cruz, Dallas TE, USA), anti-GAPDH clone 6C5 (Millipore, Mab374), anti-SIK1 (Proteintech, Chicago, IL, USA), anti-SIK3 (from Abcam, Cambridge, UK). anti-SIK2 (D28G3), anti phospho-Ser246/Ser259/SerSer155 HDAC4/5/7 (D27B5), anti-HDAC5, phospho-p38 MAPK (Thr180/Tyr182) (D3F9) XP, p38 MAPK (D13E1) XP, phospho-NF-κB p65 (Ser536), NF-κB p65 (D14E12), phospho-p44/42 MAPK (Erk1/2) (Thr202/Tyr204), p44/42 MAPK (Erk1/2), phospho-SAPK/JNK (Th183/Tyr185), SAPK/JNK (all from Cell Signaling Technology, Danvers, MA, USA).

### Statistical analysis

All results were calculated using GraphPad Prism 6.0 software (GraphPad Software, Inc., La Jolla, CA, USA) and are presented as the mean ± SD of at least three independent experiments unless indicated otherwise. A statistical comparison of two groups was performed using the Student's t test. Inter group variation was measured by one-way analysis of variance (ANOVA) followed by Dunnett’s multiple comparison test. In all cases, p < 0.05 was considered statistically significant.

## Results

### Expression of SIK1-3 in osteoclasts derived from RAW264.7 cells or BMM

**Fig 1:**
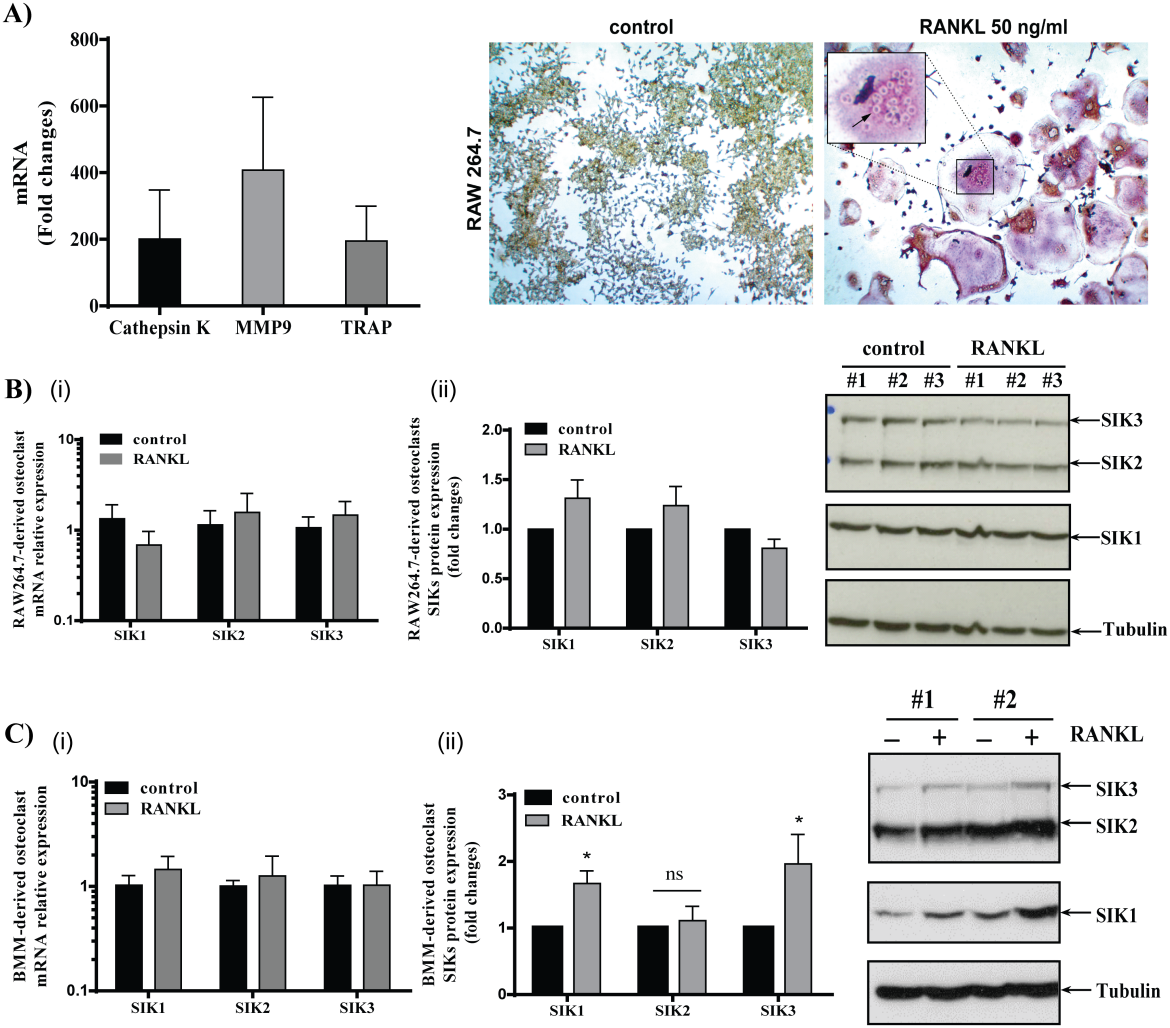
Expression of SIK1-3 in osteoclasts derived from RAW264.7 cells or BMM. RAW264.7 cells were stimulated for 4 days with RANKL (50 ng/ml) to induce differentiation into osteoclasts. **A)** mRNA expression levels of Cathepsin K, MMP9 and TRAP genes were determined by RT-qPCR using 18S rRNA as normalization control and calculated by the 2-^ΔΔ^Ct method (n = 5). Data are expressed as fold changes versus untreated RAW264.7 cells (set as one). A representative example of formation of giant multinucleated cells detected by TRAP staining is depicted. **B)** Relative SIK1, SIK2 and SIK3 mRNA expression (**i**) in RAW 264.7 cells (n = 6) following RANKL stimulation (50ng/ml, 4days). mRNA was measured by RT-qPCR using 18S rRNA as normalization control and calculated by the 2-^ΔΔ^Ct method. Protein expression levels (**ii**) of SIK1, SIK2 and SIK3 in cell lysates separated on a 7.5% SDS-PAGE (50 μg protein/lane) were determined by Western blot and normalized for tubulin expression (n = 4) **C)** Relative SIK1, SIK2 and SIK3 mRNA expression (**i**) in BMM cells (n = 7) following RANKL stimulation (50ng/ml) and MCSF (30 ng/ml), for 3 days. mRNA was measured by RT-qPCR using 18S rRNA as normalization control and calculated by the 2-^ΔΔ^Ct method. Protein expression levels (**ii**) of SIK1, SIK2 and SIK3 in cell lysates separated on a 7.5% SDS-PAGE (50 μg protein/lane) were determined by Western blot and normalized for tubulin expression (n = 5). Protein data are expressed as fold changes versus control cells (set as one). Data are expressed as mean +/- SD. Statistical significance is reported as * *P*<0.05.

Stimulation of RAW 264.7 cells with 50 ng/ml of RANKL for 4 days resulted in the formation of several giant multinucleated osteoclasts, as monitored by the tartrate-resistant acid phosphatase (TRAP) staining assay and in a significant increase of mRNA expression of osteoclast-specific genes like Cathepsin K, matrix metalloproteinase (MMP9) and TRAP (Fig 1A) [14, 15]. In these cells we analysed the expression of SIK1-3 and whether differentiation into osteoclasts would affect their expression levels. The expression of SIK1, -2 and -3 mRNA and proteins were not significantly changed in osteoclasts compared to unstimulated RAW264.7 cells (Fig 1B).

We also examined osteoclasts derived from primary BMM by treatment with M-CSF and RANKL for 3 days. SIK mRNA levels were not significantly affected by the differentiation into osteoclasts. However, SIK1 and -3 showed a modest (~2-fold), but significant increase suggesting that post-translational mechanisms regulate SIK1 and SIK3 expression in these cells (Fig 1C).

### The SIK inhibitor HG-9-91-01 reduces RANKL-induced osteoclast differentiation markers and resorption lacunae activity

**Fig 2:**
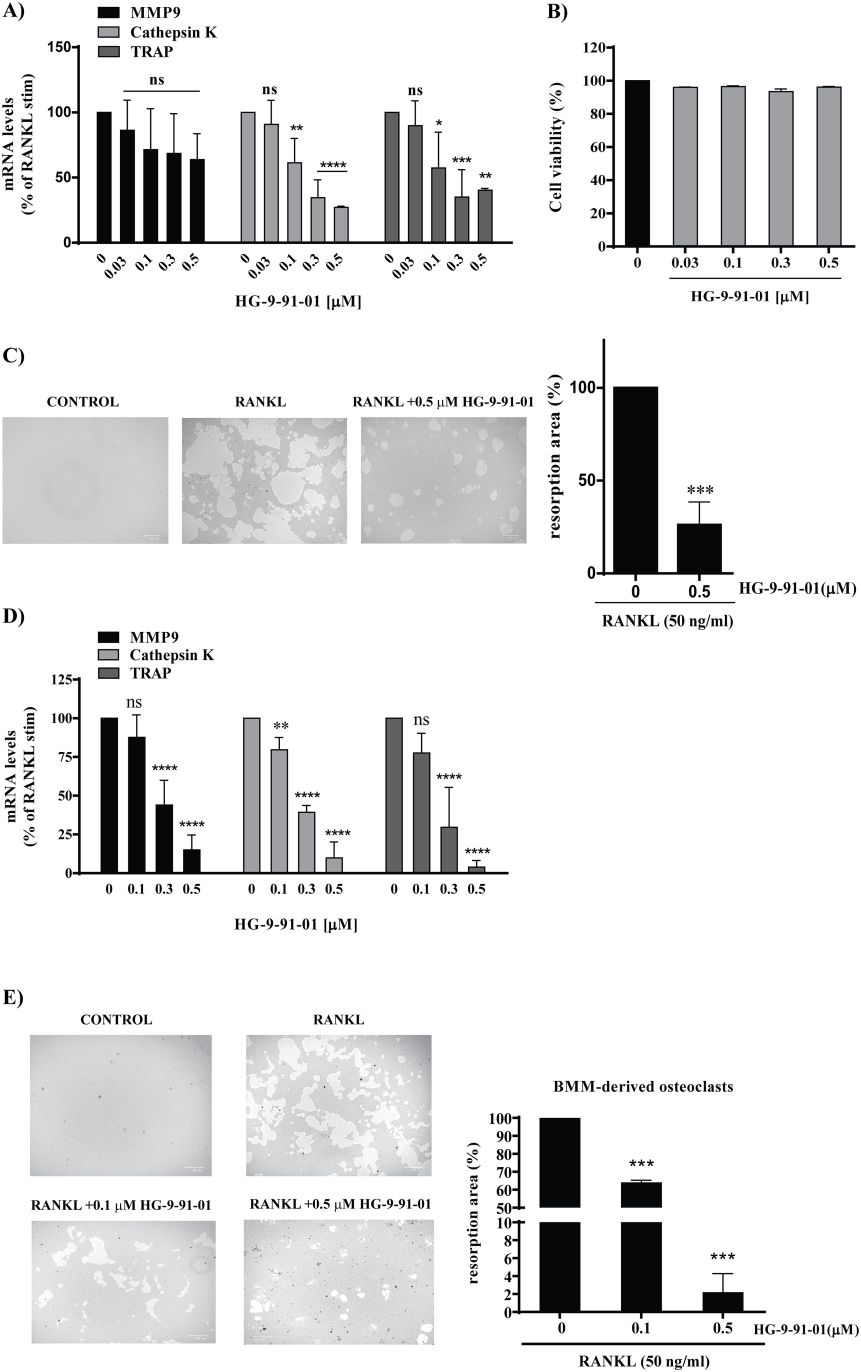
SIK inhibitor HG-9-91-01 reduces RANKL-induced osteoclast differentiation markers and bone-resorbing activity. **A**) RAW264.7 cells were preincubated with vehicle (0.03% DMSO) or indicated concentration of SIK inhibitor HG-9-91-01 for 30 min followed by RANKL (50 ng/ml) stimulation. After 4 days mRNA expression levels of MMP9, Cathepsin K, and TRAP genes were determined by RT-qPCR using 18S rRNA as normalization control (n = 5). **B)** Cell viability was determined in the supernatants by measuring LDH release. The histograms represent the levels of mRNA or LDH (%) compared with that of the RANKL control (set = 100%). **C)** RAW264.7 cells were cultured as above in 24 well Osteo plate in presence of 0.5 μM of HG-9-91-01. After 4 days the cells were removed with 5% sodium hypochlorite solution and resorbed areas were measured. The histograms represent the relative resorbed area (%) compared with that of RANKL-stimulated control (set = 100%). A representative example is depicted. **D)** BMM cells (n = 4) were preincubated with vehicle (0.03% DMSO) or the indicated concentration of SIK inhibitor HG-9-91-01 for 30 min followed by RANKL stimulation (50ng/ml) and MCSF (30 ng/ml) for 3 days. mRNA analysis was performed as above. **E)** BMM were cultured as above in 24 well Osteo plate in presence of 0.1 or 0.5 μM of HG-9-91-01. After 3 days resorbed areas were measured as above. The histograms represent the relative resorbed area (%) compared with that of RANKL-stimulated control (set = 100%). A representative example is depicted. Data are expressed as mean +/- SD. * *P*<0.05, **P<0.01, ***P<0.001 significant *vs*. RANKL control.

To test the hypothesis that SIK inhibition would have an impact on osteoclastogenesis, we pretreated RAW264.7 for 30 min with increasing concentrations of the pan-SIK inhibitor HG-9-91-01 before RANKL stimulation. As compared to untreated cells, the addition of the pan-SIK inhibitor resulted in reduction of multinucleated cell formation and of TRAP staining (*data not shown*), and significantly reduced the mRNA of osteoclast differentiation markers in a concentration dependent manner (Fig 2A). The effect of SIK inhibitor was significant for Cathepsin K and TRAP, whereas only a trend to inhibition of MMP9 was observed (Fig 2A). To rule out that the effect of HG-9-91 01 was due to unspecific cell toxicity of the compound, we performed a cell viability assay. No significant toxicity was observed at the concentrations tested (Fig 2B). We then further examined whether HG-9-91-01 has an effect on the ability of mature osteoclasts to resorb bone using resorption lacunae formation assay. RAW-derived osteoclasts were able to form a great number of lacunae on the bottom of the plate upon RANKL stimulation indicating that the mature osteoclasts were functionally active. Pre-treatment with 0.5 μM HG-9-91-01 significantly reduced the formation of resorption lacunae compared to RANKL alone. (Fig 2C).

In osteoclasts differentiated from BMM we also observed a significant concentration-dependent reduction of all three osteoclast markers (Fig 2D).

BMM-derived osteoclasts formed a great number of lacunae on the bottom of the plate upon RANKL stimulation. Pre-treatment with HG-9-91-01 greatly reduced the formation of resorption lacunae compared to RANKL alone in a concentration dependent fashion (Fig 2E).

### SIK inhibitor HG-9-91-01 does not affect MAPK and NF-κB activation in RANKL-stimulated RAW264.7 cells

**Fig 3:**
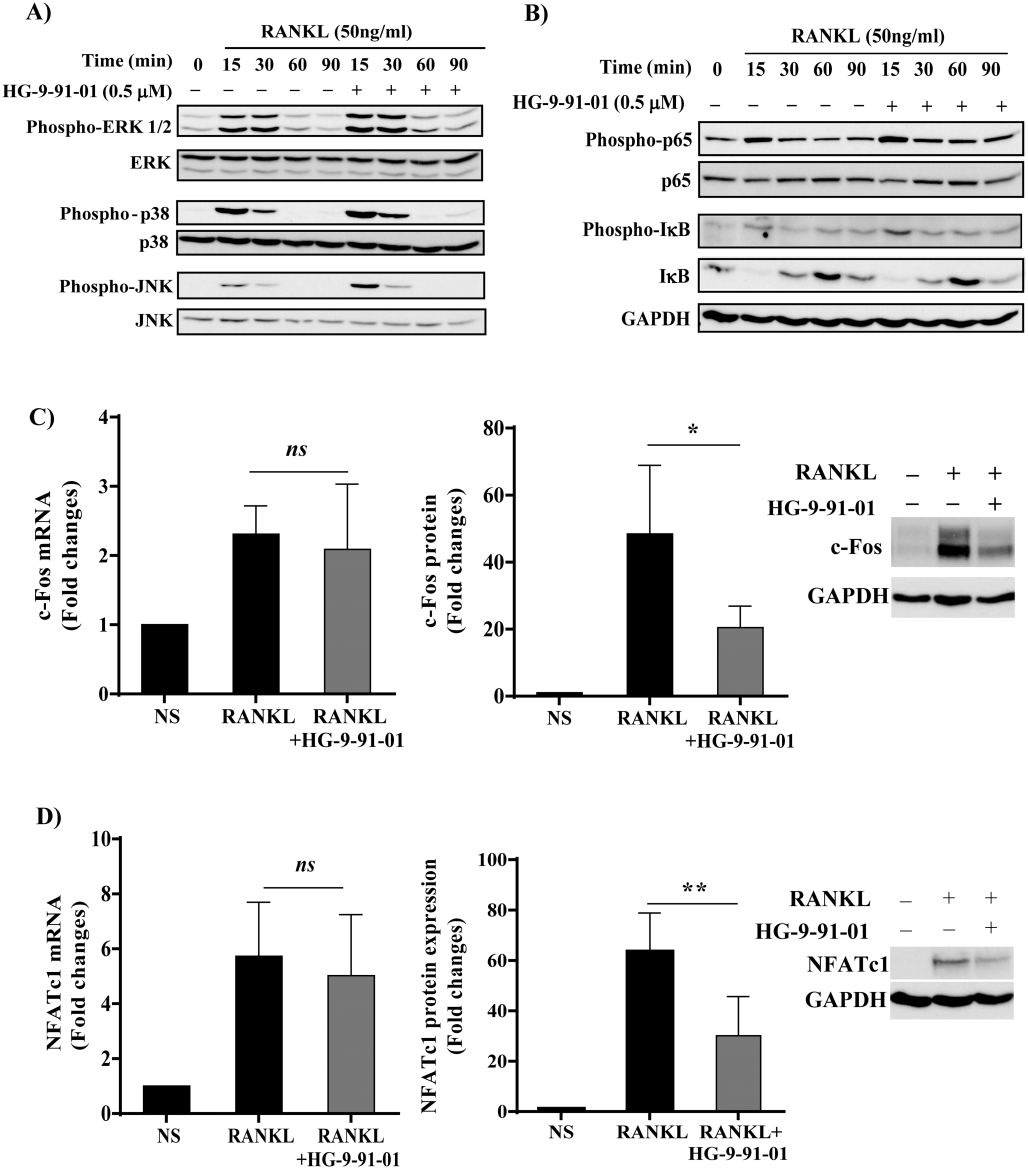
Effect of SIK inhibitor HG-9-91-01 on RANKL-induced signalling pathways. Effect of HG-9-91-01 pretreatment on NF-κB, MAPKs and JNK activation in RANKL stimulated RAW264.7 cells. Cells were cultured in 1% serum for 16h, preincubated with vehicle (0.03% DMSO) or 0.5 μM of SIK inhibitor HG-9-91-01 for 30 min followed by RANKL (50 ng/ml) stimulation for the indicated time points. Total protein extracts (25 μg/lane) were separated by 10% SDS-PAGE followed by immunoblotting using specific antibodies against **A)** phospho-ERK 1/2, phospho-p38 and phospho-JNK and **B)** phospho-p65, phospho IκB or GAPDH. Membranes were stripped and reprobed with anti ERK1/2, anti-p38, anti-JNK, anti-p65 or anti-IκB antibodies, respectively. *Insets* show a representative example of four independent experiments. Effect of HG-9-91-01 pretreatment on **(C)** c-Fos and **(D)** NFATc1 expression. RAW264.7 cells were preincubated with vehicle (0.03% DMSO) or 0.5 μM of SIK inhibitor HG-9-91-01 for 30 min followed by RANKL (50 ng/ml) stimulation for 24h. mRNA expression levels of c-Fos and NFATc1 genes were determined by RT-qPCR using 18S rRNA as normalization control (n = 9). Total protein extracts (50 μg/lane) were separated by 10% SDS-PAGE followed by immunoblotting using specific antibodies (n = 6). *Insets* show a representative example depicting c-Fos, NFATc1 and GAPDH expression. c-Fos and NFATc1 were quantified relative to GAPDH. Data are presented as fold changes versus untreated cells (set as one) and expressed as mean +/- SD. * P<0.05, **P<0.01, significant vs. RANKL-stimulated control.

To explore the pathways by which SIK inhibition regulates osteoclast differentiation we analyzed its effect on RANKL-induced activation of NF-κB and MAPK, respectively. Consistent with previous reports [9, 16, 17], a time course of RANKL stimulation of RAW 264.7 cells induced a strong phosphorylation all the three MAPK families (Fig 3A) and of p65, IκB (Fig 3B), which peaked around 15–30 min and returned to basal levels by 90 min. The preincubation with SIK inhibitor did not inhibit RANKL-induced p65 or IκB phosphorylation. Similarly, no inhibition, but rather a small increase in the phosphorylation status of ERK, JNK and p38 was observed upon HG-9-91-01 pretreatment.

### SIK inhibitor HG-9-91-01 reduces c-Fos and NFATc1 protein expression in RANKL-stimulated RAW264.7 cells

RANKL stimulation strongly induces the nuclear factor activated T cells, cytoplasmic 1 (NFATc1), which is the master transcription factor responsible for the regulation of osteoclast-specific genes, including TRAP, Cathepsin K and MMP9 [18]. c-Fos is also rapidly induced by RANKL stimulation [19] and is indispensable for osteoclast differentiation [20, 21] We determined whether HG-9-91-01 pretreatment would decrease c-Fos and/or NFATc1 levels. RAW cells stimulated with RANKL showed a 2.5-fold induction of c-Fos mRNA after 24 h, whereas c-Fos protein expression in the same time-frame increased by 48-fold. Interestingly, pretreatment with SIK inhibitor had no effect on c-Fos mRNA levels, but significantly reduced c-Fos protein levels by ~58% (Fig 3C). NFATc1 mRNA expression was induced by 6-fold after 24 h of RANKL stimulation and its protein levels were increased by ~60-fold. HG-9-91-01 treatment significantly reduced NFATc1 protein by ~53%, whereas had no effect on its transcript levels (Fig 3D). Similar effects were also confirmed in BMM-derived osteoclasts (data not shown). Overall these results are consistent with the effect of the pan-SIK inhibitor HG-9-91-01 on osteoclastogenesis and suggest that its action on c-Fos and NFATc1 occurs mainly via post-transcriptional mechanisms.

### Proteasome inhibition partially reverts the effect of SIK inhibitor on c-Fos reduction

**Fig 4:**
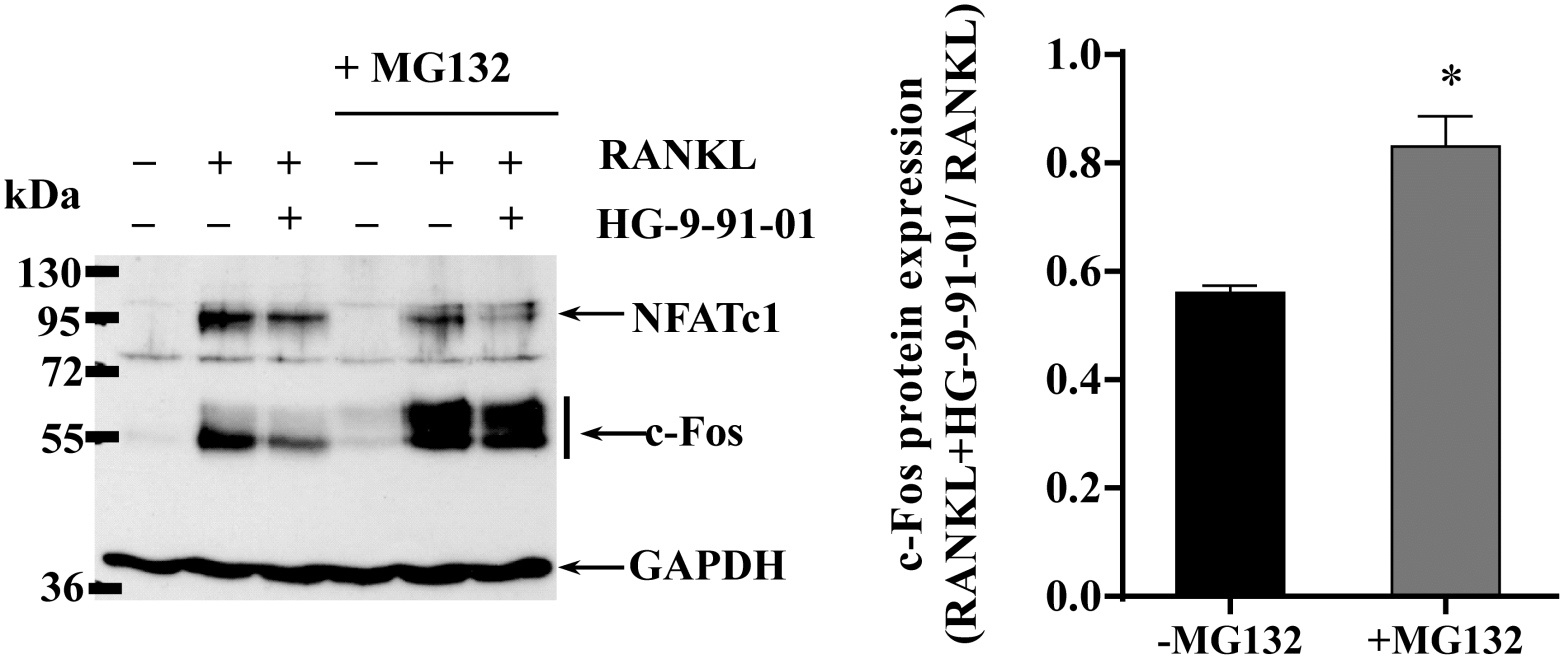
Proteasome inhibition partially reverts the effect of SIK inhibitor on c-Fos reduction. RAW264.7 cells were preincubated with vehicle (0.03% DMSO) or 0.5 μM of SIK inhibitor HG-9-91-01 for 30 min followed by RANKL (50 ng/ml) stimulation for 24h. MG132 (10 μM) was added during the last 4h of incubation. Total protein extracts (50 μg/lane) were separated by 10% SDS-PAGE followed by immunoblotting using specific antibodies. *Inset* show a representative example depicting c-Fos, NFATc1 and GAPDH. c-Fos expression levels in presence or absence of MG132 treatment were quantified relative to GAPDH and represented as ratio of their respective RANKL stimulated control (n = 3). Data are expressed as mean +/- SD. * P<0.05 *vs*. no MG132 treatment.

Our results showed that RANKL exerted marked stimulatory effects on c-Fos protein, whereas it has smaller positive effect on c-Fos mRNA levels. This finding suggests that the accumulation of c-Fos protein in osteoclasts is mainly regulated at a post-translational level. Consistently, it also plausible that SIK inhibition regulates the expression of c-Fos protein by increasing its degradation. To test this hypothesis, the proteasome inhibitor MG132 was added to cell cultures 4 hours before terminating the incubation with RANKL. MG132 revealed the presence of c-Fos ubiquitination, as suggested by the upwards shift in the molecular weight of the c-Fos protein band, and significantly reverted the inhibitory effect of HG-91-91-01 on c-Fos levels from a 58% to a 17% (p<0.05) (Fig 4).

NFATc1 levels are also known to be regulated by proteasome degradation [22, 23]. However, it seems that this mechanism is not influenced by SIK inhibition since MG132 treatment had no effect on NFATc1 levels (Fig 4).

### RANKL stimulation of RAW264.7 cells affects the LKB1/SIK-mediated signaling axis

**Fig 5:**
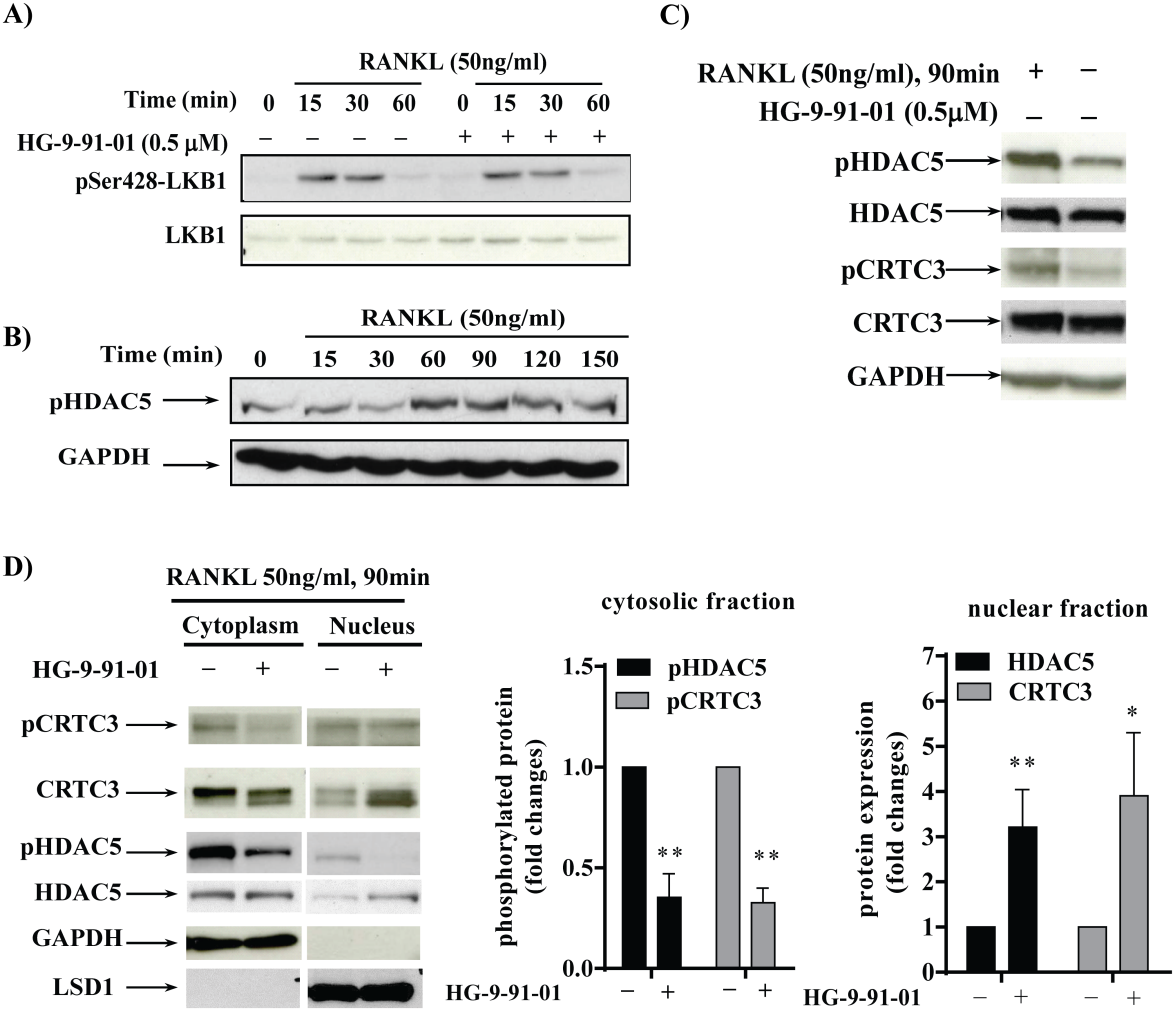
RANKL stimulation modulates the LKB1-SIK signaling axis in RAW cells. RAW264.7 cells were cultured in 1% serum for 16h, preincubated with vehicle (0.03% DMSO) or 0.5 μM of SIK inhibitor HG-9-91-01 for 30 min followed by RANKL (50 ng/ml) stimulation for the indicated time points. Total protein extracts (25 μg/lane) were separated by 10% SDS-PAGE followed by immunoblotting using specific antibodies against **A)** phospho-Ser428 LKB1 and **B)** phospho-Ser259 HDAC5. Membranes were stripped and reprobed with anti-LKB1 and GAPDH, respectively. *Insets* show a representative example of four independent experiments. **C)** Effect of HG-9-91-01 on RANKL-induced phosphorylation of HDAC5 and CRTC3. RAW264.7 cells were preincubated with vehicle (0.03% DMSO) or 0.5 μM inhibitor for 30 min followed by RANKL (50 ng/ml) stimulation for 90 min. Total protein extracts (25 μg/lane) were separated by 10% SDS-PAGE followed by immunoblotting using specific antibodies against phospho-Ser259 HDAC5, pSer370-CRTC3 and GAPDH. Membranes were stripped and reprobed with anti-HDAC5, anti-CRTC3, respectively. *Inset* shows a representative example of three independent experiments. **D)** Effect of HG-9-91-01 on cytosolic and nuclear redistribution of HDAC5 and CRTC3 upon RANKL stimulation. RAW264.7 cells were preincubated with vehicle (0.03% DMSO, control) or 0.5 μM HG-9-91-01 for 30 min followed by RANKL (50 ng/ml) stimulation for 90 min. Cytosolic and nuclear proteins extracts were prepared as detailed in *Material and Methods*. Fifty μg/lane were separated by 10% SDS-PAGE followed by immunoblotting using specific antibodies against phospho-Ser259 HDAC5 and phosphoSer370-CRTC3. Membranes were stripped and reprobed with anti-HDAC5 and CRTC3. Probing with LSD1 and GAPDH antibodies was used as qualitative control of the nuclear and cytosolic preparations, respectively. Data are presented as fold changes versus control cells (set as one) and represent the mean +/- SD. *Inset* shows a representative example of three independent experiments. * P<0.05, **P<0.01.

Having established that SIK kinase activity has an impact on osteclastogenesis, we examined the effect of RANKL stimulation on the proteins which are known to signal upstream and downstream of SIK kinases. Liver kinase B1 (LKB1) is the master kinase which phosphorylates and activates SIK and all the other members of AMPK family [24]. Upon RANKL stimulation the phosphorylation of Ser428-LKB1 increased after 15 and 30 min and returned to basal levels by 60 min (Fig 5A). As expected no effect of the SIK inhibitor was observed on pSer428-LKB1. We also examined the effect of RANKL stimulation towards the phosphorylation of HDAC5, a class II histone deacetylase known to be a SIK substrate [25]. We showed that phosphorylation of pSer259-HDAC5 increased after 60–90 min and remained elevated up to 150 min after RANKL stimulation (Fig 5B). Moreover, we confirmed that the inhibitory effects of HG-9-91-01 on c-Fos and NFATc1 correlated with the dephosphorylation of SIK substrates. RAW cells pre-treated with HG-9-91-01 before RANKL stimulation showed a reduced phosphorylation of HDAC5 and of the CREB regulated transcription co-activator 3 (CRTC3), another known SIK substrate [2] (Fig 5C). We next verified whether SIK inhibition increases HDAC5 and CRTC3 nuclear translocation, which is known to occur upon their dephosphorylation [26]. Nuclear and cytosolic extracts of RAW cells were prepared after 90 min of RANKL-stimulation in absence or presence of HG-9-91-01 and analyzed by Western blot. We demonstrated that levels of HDAC5 in the nuclear fractions were enriched by 3-fold in HG9-91-01-treated cells compared to RANKL-stimulated cells in absence of SIK inhibitor and that a similar effect occurs for CRTC3 (Fig 5D). Overall these results suggest that RANKL stimulation has an impact of the activation of LKB1/SIK signaling axis.

### CRISPR/Cas9-mediated SIK 2 and 3 knock-down reduces c-Fos and NFATc1 expression upon RANKL stimulation and impairs osteoclast formation

**Fig 6:**
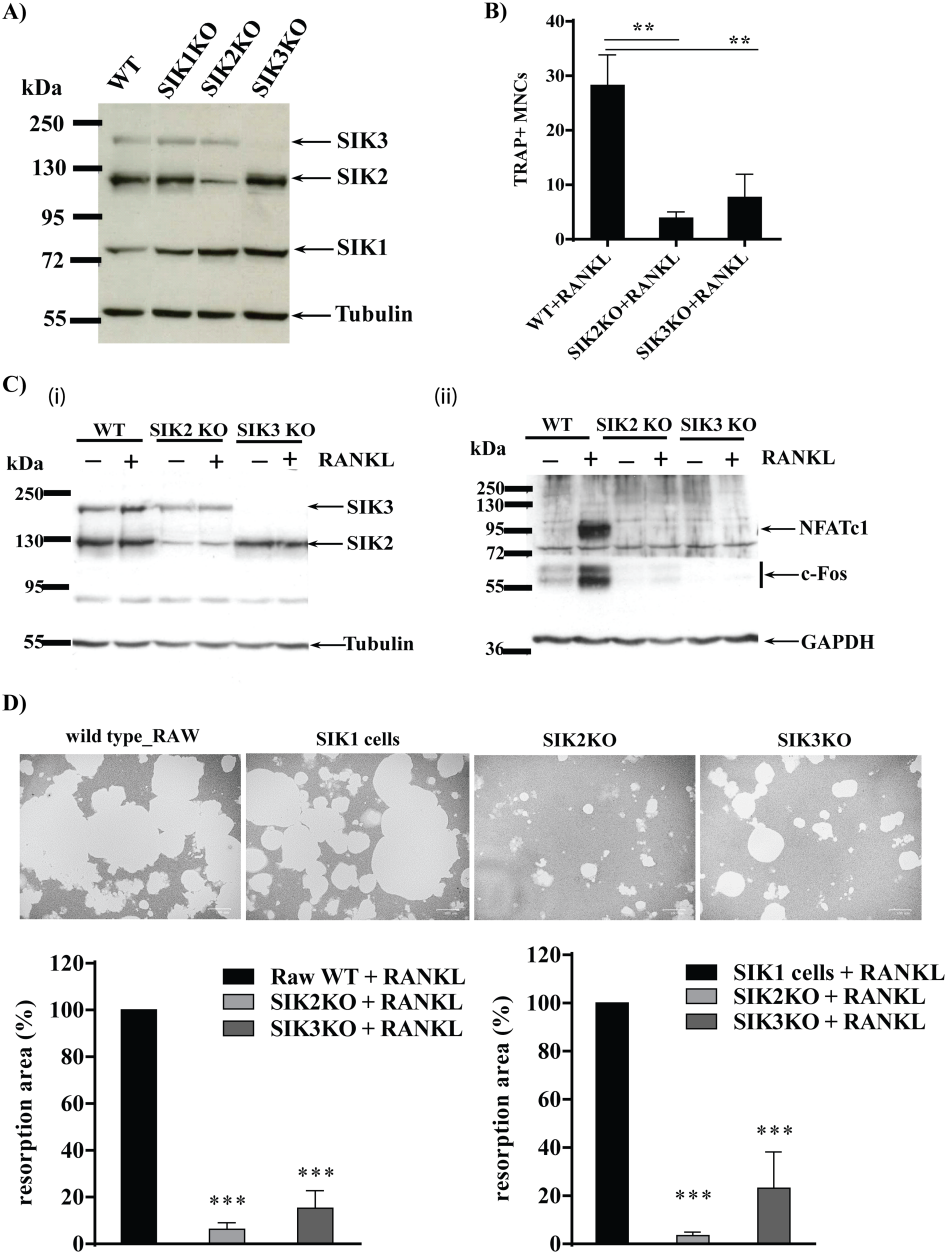
SIK2 or -3 silencing by CRISPR/Cas9 mediated gene editing reduces osteoclast differentiation and impairs resorption activity upon RANKL stimulation. **A)** RAW264.7 cells were infected with pLentiCRISPR-V2 viruses targeting SIK1, SIK2, and SIK3, respectively as detailed in *Material and Methods* and SIK expression was determined in cell lysates (25 μg/lane) form RAW 264.7 infected cells or wild type cells separated by 7.5% PAGE-SDS and probed with specific antibodies and anti-tubulin. A representative example is depicted (n = 3). **B**) SIK KO or wild type RAW cells were incubated with 50 ng/ml RANKL for 4 days and then were fixed and stained with TRAP. The TRAP positive multinucleated cells (TRAP+ MNCs) were visualized by digital microphotography and the TRAP+ MNCs containing more than 3 nuclei from four randomly selected magnification fields (scale bar 100 μm) were counted. Error bars = mean +/- SD, from one independent experiment performed in triplicate. **C**) Cells Lysates obtained from SIK KO cells or wild type RAW 264.7 cells cultured in absence or presence of RANKL (50 ng/ml) for 24 h were separated by 7.5% PAGE-SDS and SIK expression determined as above (**i**), in the same lysates separated on 10% PAGE-SDS (50 μg/lane) the expression of c-Fos and NFATC1 were determined (**ii**). GAPDH expression was used as loading control. A representative example (n = 3) is depicted. **D)** SIK KO cell or wild type RAW were cultured as above in 24 well osteo plates. After 4 days the cells were removed with 5% sodium hypochlorite solution and resorbed areas were measured. Four randomly selected magnification fields (scale bar 100 μm) for each condition were analyzed. The histograms represent the relative resorbed area (%) compared with that of wild type RAW or SIK1 control (set = 100%). Data are expressed as mean +/- SD, from one experiment performed in triplicate. Data are representative of two independent experiments. *Insets* show a representative example for each condition. * P<0.05, **P<0.01, ***P<0.001.

To complement the observations obtained using SIK inhibitors and get insights on the role of individual SIK in osteoclastogenesis, we selectively knocked-out each individual SIK by CRISPR/Cas9 gene editing. Despite the use of two different gRNA sequences for *SIK1*, we could not achieve a significant reduction of SIK1 protein levels. We then used these cells as additional negative control (“SIK1 cells”). In contrast, SIK2 and SIK3 were successfully and specifically knocked down (~75% for SIK2 KO and ~90% for SIK3 KO, respectively) in bulk cultures obtained upon puromycine selection (Fig 6A). When the SIK2 KO and SIK3 KO cells were stimulated with RANKL, this resulted in a significant reduction of differentiated osteoclasts measured by TRAP staining (Fig 6B). As consequence of SIK2 or SIK3 protein knock-out (Fig 6C, **left panel**) the RANKL-induced c-Fos and NFATc1 protein expression were completely abolished compared to wild-type RAW cells (Fig 6C, **right panel**). Moreover, we observed a greatly reduced formation of resorption lacunae in SIK2 KO and, to a lesser extent, in SIK3 KO compared to either wild-type RAW or control “SIK1 cells” (Fig 6D).

## Discussion

In this study we analyzed the expression and the function of SIK in osteoclastogenesis and demonstrated that the SIK inhibitor HG-9-91-01 dose dependently reduces RANKL-induced mRNA expression of osteoclast differentiation markers (i.e. Cathepsin K, MMP-9 and TRAP). Consequently, SIK inhibition impacts significantly bone resorbing activity by greatly reducing bone pits formation in RANKL-stimulated RAW 264.7 cells and in primary BMM. Our results strongly support the notion that SIK play a significant role among the check-points controlling osteoclastogenesis. The analysis of the molecular pathways activated by RANKL upon treatment with SIK inhibitor allowed us to elucidate the molecular mechanisms of the effect of SIK inhibition. The RANKL-mediated activation of NF-κB and MAPK families (p38, ERK and JNK) signalling molecules induces mature and functionally active osteoclasts by upregulating the transcription factors c-Fos and NFATc1[7]. The role of c-Fos in osteoclastogenesis has been confirmed in c-Fos knock-out mice, which develop osteopetrosis due to impaired osteoclast formation [21]. c-Fos complexes with c-Jun to form the AP-1 transcription factor, which is essential for NFATc1 expression [27]. NFATc1 is a master transcription factor involved in the terminal differentiation of osteoclasts and its transcriptional induction is also dependent on NF-κB. In addition, NFATc1 is responsible of its own transcription [18]. We established that SIK inhibition is devoid of any inhibitory effect on RANKL-mediated NF-κB and MAPK activation and on the transcriptional regulation of c-Fos and NFATc1, but significantly reduced c-Fos and NFATc1 protein levels.

In agreement with a previous report [28], we demonstrated that RANKL-mediated increase in c-Fos expression shows higher amplitude of induction in protein compared to mRNA expression, suggesting that a post-translational mechanism is mainly responsible for c-Fos protein accumulation in differentiating osteoclasts. Ito et al. also established that the stability of c-Fos in osteoclast progenitor cells is regulated by the ubiquitin-proteasome dependent degradation of c-Fos. We demonstrated that proteasome inhibition reverts partially the effect of SIK inhibitor, suggesting that additional mechanism(s) contribute to the effect of SIK inhibition on c-Fos expression.

NFATc1 levels are also known to be regulated by proteasome degradation as part of a self-limiting regulatory loop that controls osteoclastogenesis. NFATc1 proteins are ubiquitinated and rapidly degraded during the late stage of osteoclast formation, a process that is mediated by Cbl-b and c-Cbl ubiquitin ligases in a Src-dependent [22] or Nurr77-dependent manner[23]. We demonstrated that a posttranslational mechanism is responsible for the effect of SIK inhibitor on NFATc1. However, proteasome inhibition does not impact the effect of SIK inhibition on NFATc1. We also tested whether SIK inhibition affected Nurr77-mediated NFATc1 degradation by using BMM-derived osteoclasts from Nurr77 KO mice. The effect of SIK inhibitor on osteoclast-specific markers was the same in cells from Nurr77 KO mice and wild-type littermates (*data not shown*), indicating that SIK inhibition has no influence on Nurr77-mediated NFATc1 degradation. Thus, the exact mechanism by which SIK inhibition reduces NFATc1 remains to be elucidated.

According to the currently accepted model, tonic SIK activity is maintained by the “master kinase” LKB1, which phosphorylates SIK and other members of the AMPK kinase family at a conserved Thr residue [24]. LKB1 itself can be phosphorylated and activated at multiple sites by different kinases and this could potentially alter its *de novo* activity and ability to interact with its substrates [29]. Notably, when LKB1 is phosphorylated at Ser428, its ability to activate AMPK-kinase family members, including SIK, is increased [30]. We have previously demonstrated that in human macrophages pro-inflammatory stimuli such LPS and IL-1β induce a rapid and transient LKB1 phosphorylation at Ser428 [5]. Interestingly, we demonstrated here that a similar increase in LKB1 phosphorylation occurs upon RANKL-stimulation. As consequence, this suggests that SIK activity towards its substrates is increased by RANKL stimulation as demonstrated by the increased phosphorylation of HDAC5, a known substrate of SIK.

The data we obtained with RAW cells, in which SIK2 and -3 expression was greatly reduced by CRISPR-CAS9-mediated gene editing, confirm that the effects observed with SIK inhibitor is specific to SIK inhibition and not influenced by potential off-target mechanisms of the compound. In addition, these experiments allowed us to establish that SIK2 and, to a lesser extent SIK3, are likely the kinases involved in controlling osteoclast formation and suggest a partial redundancy between these two kinases. Global SIK2 KO mice do not show skeletal phenotype, but SIK3 KO mice have severely malformed skeletons and most die at birth [31]. However, a role of SIK1 cannot be completely excluded since, despite several attempts with different gRNA targeting various regions of the SIK1 gene, we could not effectively target the expression of SIK1 in RAW cells. A possible explanation is that the SIK1 gene in RAW cells is poorly transcriptionally active and thus is not easily accessible to the CRISPR-CAS9 machinery. This is supported by previous observations made by us (Lombardi et al. *unpublished observation*) and other authors demonstrating that in RAW cells SIK1 mRNA expression is ~100-fold lower than SIK2 and -3 [2] and its expression could only be partially (~20%) knocked out by transient RNAi in human macrophages [5]. Alternatively, it is possible that SIK1 expression is indispensable for RAW cells survival.

A recent study described the generation of SIK1, -2 and -3 kinase-dead knock-in mice and addressed the role of kinase dependent and independent functions of each SIK in macrophage development and polarization [32]. Generation of osteoclasts derived from BMM of these kinase-dead knock-in mice compared to wild-type BMM would represent a valuable tool to address the specific role of each SIK activity in osteoclastogenesis.

The role of SIK2 and 3 in bone formation has been recently investigated in osteocytes in which it has been demonstrated that SIK inhibitors *in vitro* can mimic the effect of parathyroid hormone (PTH), the only approved osteoporosis treatment that stimulates new bone formation. Treatment of mice with the SIK inhibitor YKL-05-099, an analogue of HG-9-91-01 with a PK profile suitable for *in vivo* use, increases bone formation and bone mass [8]. Parathyroid hormone is also increasing osteoclastic bone resorption, in part due to PTH-induced RANKL up-regulation in osteocytes [33]. However, *in vivo* treatment with the SIK inhibitor YKL-05-099 actually decreased osteoclast numbers [8]. This effect of the SIK inhibitor is thus in agreement with our results. The authors attributed it to a partial inhibition by YKL-05-099 of the tyrosine kinase Src whose deficiency leads to functional osteoclast defects and osteopetrosis [34]. However, our current study investigating in more detail the role of SIK in osteoclastogenesis and our data reproducing the effect of SIK inhibition in the SIK2 and -3 KO cells suggest that the effect of SIK is specific and not due to potential off-target Src inhibition. Therefore, it is tempting to speculate that SIK activities play a role in fine tuning the balance between bone formation and bone resorption by their function in osteocytes and osteoclasts, respectively.

Excessive osteoclasts activation can lead to osteolysis, which is a key feature in several inflammatory pathologies such as rheumatoid arthritis and various bone diseases including osteoporosis, periodontitis, Paget’s disease and osteolytic tumors [35, 36] Current therapies are targeting either osteoclasts (i.e. antiresorptive bisphosphonates or Cathepsin K inhibitors) or osteoblasts (i.e. anabolic parathyroid hormone) but are not spared of sometimes severe side effects (e.g. renal toxicity and osteonecrosis). SIK inhibition could thus potentially represent a novel therapeutic approach aiming at inhibiting bone resorption.

## Supporting information

S1 File Supplementary methods. (PDF)

S1 Fig Supplementary figures raw data set western blots. (PDF)
