# Supplementary material for: Salt-inducible kinases (SIK) inhibition reduces RANKL-induced osteoclastogenesis
Source: PLoS One. 2017 Oct 3;12(10):e0185426. doi: 10.1371/journal.pone.0185426 (PMC5626034; doi:10.1371/journal.pone.0185426)
Supplement: S1 File — (PDF) [file pone.0185426.s001.pdf]

Supplementary methods

Method for CRISPR/Cas9 knock-out of SIK1, SIK2 and SIK3

To create specific SIK knock outs, oligos (Eurofins Genomics, Ebersberg, Germany) were designed using the "Optimized CRISPR design" tool [1, 2, 3]. Due to alternative splicing, there are three SIK3 isoforms. Therefore the oligos for SIK3 were designed to target the fourth exon, ensuring knock out of each isoform of SIK3. Oligo sequences are provided in **Table 2**.

The LenticrisprV2 plasmid (a kind gift of Prof. Fabio Martinon, UNIL, Lausanne, Switzerland) was recovered from Whatman filter paper in TE buffer [10 mM Tris pH 8.0, 1 mM EDTA], and used to transform Endura Chemically Competent cells (Lucigen Corporation, Middleton, WI, USA). Subsequently, a miniprep was prepared using the JETQUICK plasmid miniprep spin kit (Genomed, Nea Smirni, Greece), according to manufacturer's instructions. The LentiCRISPRv2 vector was digested by BsmBI and dephosphorylated through FastAP (both Thermo Fisher Scientific, Waltham, MA, USA). The digested vector together with a non-digested vector as control was loaded on a 0.8% agarose gel and was extracted from the gel using the QIAquick gel extraction kit (Qiagen, Hilden, Germany), according to the manufacturer's instructions. The oligo pairs for each SIK were resuspended at 100 µM and phosphorylated and annealed by T4 Ligation Buffer and T4 Polynucleotide kinase (New England Biolabs, Ipswich, MA, USA) in a thermocycler for 30 minutes at 37°C, 5 minutes at 95 °C and next ramped down at 5°C per minute. The annealed oligos were ligated in the digested vector using Quick ligase (New England Biolabs, Ipswich, MA, USA) and incubated for 10 minutes at room temperature. Thereafter, the vector containing the annealed oligos was transformed in Endura Chemically Competent cells according to manufacturer's instructions. After overnight incubation on LB Lennox plates supplemented with 100 µg/mL Ampicillin (Invitrogen, Waltham, MA, USA) plates at 37°C, a single colony was incubated overnight in LB miller medium supplemented with Ampicillin at 250 rpm at 37°C. Mini or midi prep preparation using JETQUICK plasmid miniprep or midiprep spin kit (Genomed, Nea Smirni, Greece), followed by sequencing using Sanger method to verify the oligo insert (Fasteris, Geneva, Switzerland). DNA concentrations were measured using the Nanodrop 2000C spectrophotometer (Thermo Fisher Scientific, Waltham, MA, USA).

Lentiviral infection of RAW264.7 cells

Lentivirus for SIK1, 2, or 3 KO was prepared with 40 µg of lentiCRISPRv2 vector containing the annealed oligos, by co-transfection with the packaging plasmids pVSVg and psPAX2 (both from AddGene, Cambridge, MA, USA) into HEK293T cells using transfection reagent PEI (UNIL,

Lausanne, Switzerland). As a “scrambled” control for viral infection, HEK293T cells were also transfected with 40 μg of CMV-EGFP Luciferase vector (AddGene, Cambridge, MA, USA). One day after transfection the medium was replaced for Opti-MEM 1% FBS (Thermo Fisher Scientific, Waltham, MA, USA). After 24 hours, supernatant was harvested and medium was refreshed. The procedure was repeated the next day and total supernatant was filtered through a 0.45 μM syringe filter and incubated overnight at 4 °C on a rotating wheel in a mixture with PBS, sterile NaCl and PEG. The viruses were harvested and concentrated through centrifugation and the pellet was resuspended in 200 μL sterile PBS.

RAW264.7 cells were transfected with lentiviral preparation of SIK1, SIK2, SIK3 KO or CMV-EGFP Luciferase by using polybrene (8 μg/mL, a kind gift of prof. Julien Bertran, Univeristy of Geneva, Switzerland) in DMEM containing 10% FBS. Non-infected RAW264.7 cells were used as a negative control. One day after transfection, medium was refreshed with DMEM 10% FBS without polybrene. Selection of infected cells was started 48 hours after transfection in DMEM 10% FBS supplemented with 3 μg/mL puromycin (Santa Cruz Biotechnology, Dallas, TX, USA), as determined by a puromycin titration experiment in RAW264.7 cells. After 2 weeks of selection in puromycin, the RAW264.7 knock out cell line was considered stable. Cells were passaged when confluent.

Single cell cloning

RAW264.7 SIK knock out bulk was diluted to single cell level to select single cell clones. Serial dilutions were made in a 96 wells plate. In well A1 two thousand cells were plated and two-fold serial dilutions were made, first in vertical manner and subsequently in horizontal direction. Single cell colonies were visible after about 8 days. When cells reached confluence, the single cell colonies were plated in 12-well plates for clone selection. Subsequently, SIK knock-out was assessed in the cell lysates using Western blot.

References to Methods

1. http://crispr/mit/edu

2. Sanjana, N.E. *et al*, Improved lentiviral vectors and genome-wide libraries for CRISPR screening, Nature Methods. 2014; 11 (8):783-4

3. Shalem, O. *et al*, Genome-scale CRISPR-Cas9 knockout screening in human cells, Science. 2014; 43(6166):84-87.
