# Supplementary figures and images for: Salt-inducible kinases (SIK) inhibition reduces RANKL-induced osteoclastogenesis
Source: PLoS One. 2017 Oct 3;12(10):e0185426. doi: 10.1371/journal.pone.0185426 (PMC5626034; doi:10.1371/journal.pone.0185426)

Fig 1B western

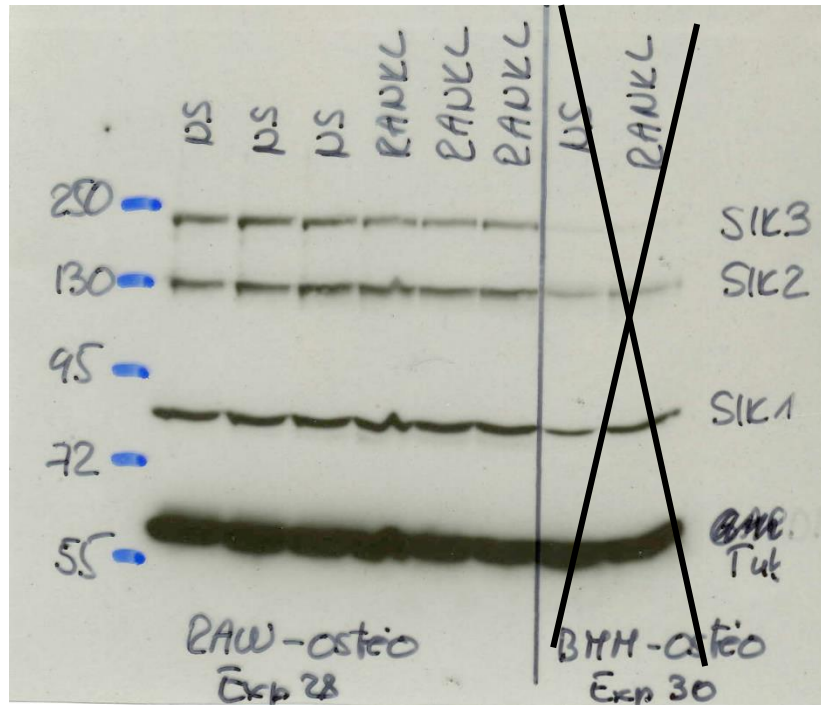

Fig 1C western

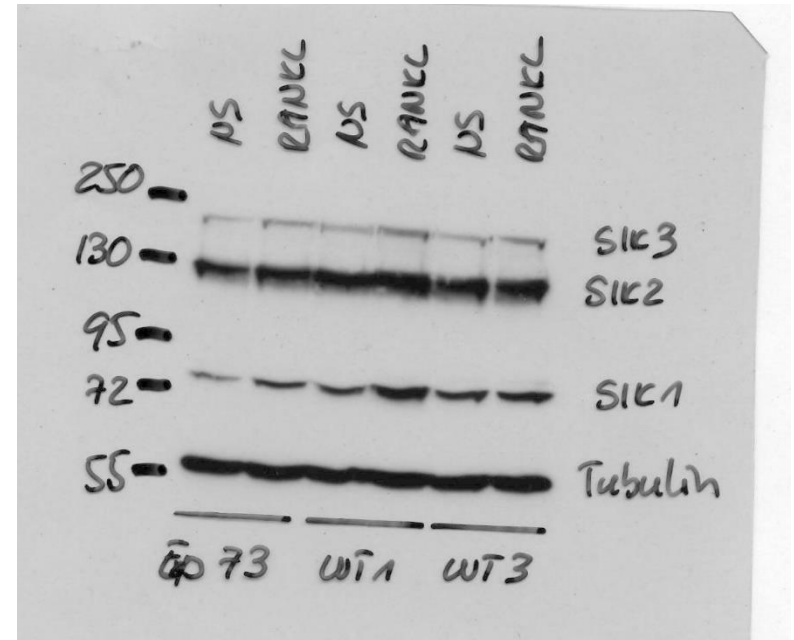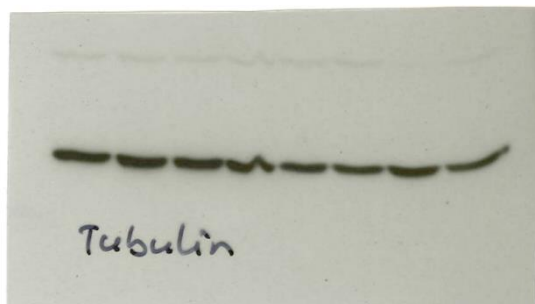

Lower exposure tubulin



Fig 5A western blots

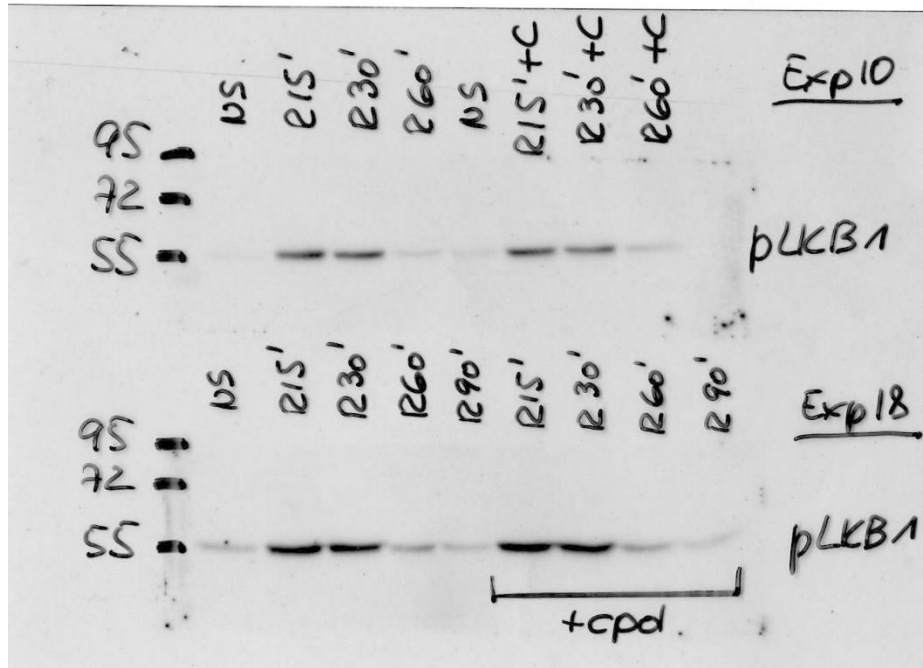

phosphoproteins

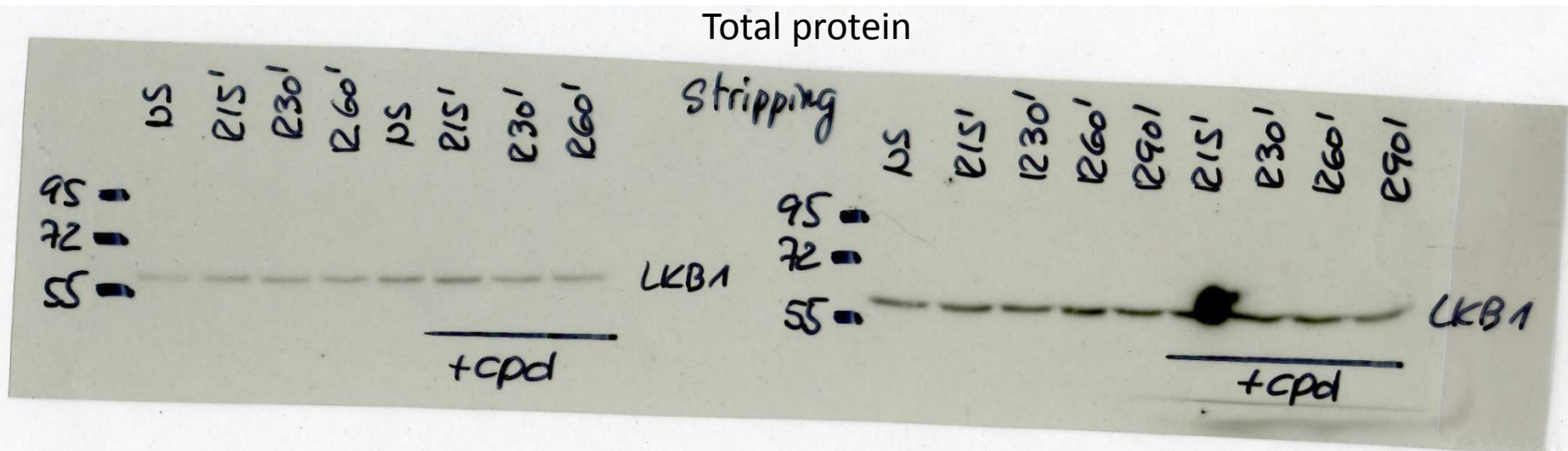

Supplement: S1 Fig — (PDF) [file pone.0185426.s002.pdf]
